# S5, a Withanolide Isolated from *Physalis Pubescens* L., Induces G2/M Cell Cycle Arrest via the EGFR/P38 Pathway in Human Melanoma A375 Cells

**DOI:** 10.3390/molecules23123175

**Published:** 2018-12-01

**Authors:** Yuqi Fan, Yiwei Mao, Shijie Cao, Guiyang Xia, Qiang Zhang, Hongyang Zhang, Feng Qiu, Ning Kang

**Affiliations:** 1 Department of Biochemistry, School of Integrative Medicine, Tianjin University of Traditional Chinese Medicine, Tianjin 300193, China; m13132215207@163.com (Y.F.); zhangqiang7@hotmail.com (Q.Z.); red318@126.com (H.Z.); 2 Tianjin State Key Laboratory of Modern Chinese Medicine, Tianjin University of Traditional Chinese Medicine, Tianjin 300193, China; 18702228257@163.com (Y.M.); caoshijie@tjutcm.edu.cn (S.C.); nyxiaguiyang@163.com (G.X.); 3 Department of Pharmaceutical Chemistry, School of Chinese Materia Medica, Tianjin University of Traditional Chinese Medicine, Tianjin 300193, China

**Keywords:** S5, G2/M arrest, EGFR, P38, melanoma

## Abstract

S5 is a withanolide natural product isolated from *Physalis pubescens* L. Our previous experimental studies found that it has significant antitumor activity on renal cell carcinoma. In the present study, the anti-melanoma effect of S5 and the related molecular mechanism was first investigated. It was found that S5 induced an obvious growth inhibitory effect on human melanoma A375 cells with low toxicity to human peripheral blood cells. Furthermore, the results demonstrated that the cell death mode of S5 on A375 cells is not due to inducing apoptosis and autophagy. However, there was a significant time-dependent increase in G2/M phase after treatment of A375 with S5. Meanwhile, S5 could also decrease the protein expression of Cdc25c, Cdc2, and CyclinB1, and increased the expression of p-P53 and P21, suggesting that S5 inhibited A375 cell death through G2/M phase arrest. Moreover, the signal pathway factors P38, extracellular regulated protein kinases (ERK), and epidermal growth factor receptor (EGFR) were observed taking part in the S5-induced A375 cells growth inhibitory effect. In addition, suppressing P38 and EGFR reversed the cell proliferation inhibitory effect and G2/M cell cycle arrest induced by S5 and inhibition of EGFR enhanced the downregulation of the expression of P38 and p-P38, indicating that S5 induced A375 G2/M arrest through the EGFR/P38 pathway. Briefly, this study explained for the first time the mechanism of S5-induced A375 cell growth inhibition in order to provide the basis for its clinical application in melanoma.

## 1. Introduction

Cutaneous melanoma, the malignant tumor of melanocytes, is the skin cancer with the highest mortality and increasing incidence worldwide [1]. Incidence of malignant cutaneous melanoma is rising especially steeply, with minimal progress in non-surgical treatment of advanced disease [2,3]. The most common chemotherapy drug presently used for melanoma is dacarbazine (DTIC) at present, which is a U.S. Food and Drug Administration (FDA)-approved, first-line treatment for patients with wild-type melanomas [4]. However, in recent years, some clinical studies show that there is an objective response rate ranging from 7%–13% when DTIC was used [5,6]. Several other chemotherapeutic agents, such as fotemustine, vindesine, and temozolomide, have similar activities comparable to DTIC [7]. Therefore, a high-efficiency drug is urgently needed for melanoma.

Apoptosis and autophagy are two evolutionarily conserved processes that maintain homeostasis during stress [8]. Apoptosis is an active, specialized form of cell death, and is well-orchestrated by a set of hierarchical molecular events [9]. Apoptosis is characterized by cell membrane blebbing, cell shrinkage, nuclear fragmentation, chromatin condensation, and chromosomal DNA fragmentation [10,11]. Caspase-3 is one of the hallmarks of apoptosis [12,13], and it is indispensable for apoptotic chromatin condensation and DNA fragmentation in all cell types examined [14]. PARP (poly ADP-ribose polymerase) is a kind of nuclear enzyme in response to DNA strand breaks. It is a cleaved substrate of caspase 3 [15]. During almost all forms of apoptosis, PARP is cleaved by caspases, suggesting that its inactivation plays the crucial role of apoptosis [16]. Autophagy is an evolutionarily conserved catabolic process in which intracellular membrane structures package dysfunctional protein complexes or organelles to degrade, and then renew these cytoplasmic components. It is thus critical for cellular integrity, intracellular homeostasis, and cell survival [17]. Autophagy is one of the adaptive mechanisms induced in melanoma cells in response to chemotherapy [18]. Inhibition of autophagy might help increase the efficiency of standard therapy to melanoma [19]. LC3 (microtubule-associated protein 1 light chain 3) is an important autophagic marker [20].

Cell cycle control is the main regulatory mechanism of cell growth. It consists of G1, S, G2, and M phases. Dysregulation of the cell cycle is one of the main features of melanoma, the dynamic interplay between cyclins, and their associated cyclin-dependent kinases (CDKs) are responsible for key transitions in the cell cycle [21]. Different cyclin-CDK complexes are activated at different points in the cell cycle [22]. For example, in the G2/M stage, the cyclin-dependent kinase CDK1 (also known as Cdc2) is thought to be the trigger of cell mitosis and when it binds to its regulatory subunit cyclin B, they can promote cell cycle entry into G2/M phase [23]. Before mitosis, cyclin B-Cdc2 complexes are held in an inactive state by phosphorylation of Cdc2 at Thr14 and Tyr15, and the dephosphorylation is carried out by the dual-specificity protein phosphatase Cdc25C. In addition, CDK activity can be counteracted by cell cycle inhibitory proteins, called CDK inhibitors (CKI), which can bind to CDK alone or to the CDK-cyclin complex and inhibit CDK activity. Two different CKI families have been discovered for now, the INK4 family and Cip/Kip family [24]. The INK family includes p15, p16, p18, and p19, which specifically inactivate CDK4 and CDK6 [25]. Furthermore, the another CKI family, Cip/Kip family, includes p21, p27, and p57, these inhibitors mainly inhibit the CDK1-cyclin B complexes [26,27]. The expression of *p21* is under transcriptional control of the tumor suppressor p53. The *p21* gene promoter contains a p53-binding site that allows p53 to transcriptionally activate *p21* [28].

Mitogen-activated protein kinases (MAPKs) are protein Ser/Thr kinases that convert extracellular stimuli into a wide range of cellular responses. The family of MAPKs include the extracellular regulated kinases (ERKs), the C-Jun N-terminal kinases (JNKs), and the p38 MAPKs [29]. The Ras-dependent ERK1/2 signal transduction pathway is a classical MAPK signal pathway, which plays an indispensable role in cell proliferation control. In normal cells, keeping activation of ERK1/2 is necessary for G1 to S phase progression and is related with induction of positive regulation of the cell cycle and inactivation of antiproliferative genes [30]. The JNK and p38 MAPK kinase pathways can be activated by a wide range of cellular stress and extracellular stimuli. Furthermore, they have been implicated in the apoptotic response of cells exposed to stress [31]. The p38 MAPK has also been verified to be associated with the cell cycle G2/M arrest [32].

The epidermal growth factor receptor (EGFR) is a tyrosine kinase receptor of the ErbB family, and it is overexpressed in a lot of malignancies [33]. Moreover, the overexpression of EGFR has been verified to promote tumor growth and progression, including maturation, angiogenesis, invasion, metastasis, and inhibition of apoptosis [34]. In human melanoma, EGFR plays a key role in its growth. It has been reported that EGFR is highly-expressed in melanoma, and its expression level is positively correlated with tumor progression and poor prognosis [35], hence it might be a useful target to inhibit melanoma via inhibiting the expression of EGFR.

S5 is a withanolide natural product isolated from *Physalis pubescens* L., which is a plant that produces nutritious and healthy fruits, named as husk tomato or hairy ground cherry. In our previous study, we found that it has a significant anti-tumor activity on renal cell carcinoma [36]. Herein, we elucidated that S5 could markedly inhibit A375 cell proliferation and it has lower cytotoxicity to human peripheral blood cells. Moreover, we report for the first time that S5 induces G2/M phase cell cycle arrest in A375 cells and the molecular mechanism of it might be mediated via the EGFR/P38 signaling pathway.

## 2. Results

### 2.1. The Effects of S5 on A375 Cell Proliferation

**Figure 1:**
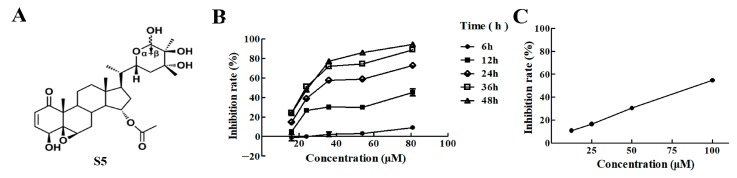
S5 inhibits the growth of A375 cells. (**A**) The structure of S5. (**B**) Inhibitory effects of S5 on cell proliferation in A375 cells. The cells were treated with 16, 24, 36, 54, and 81 μM of S5 for the indicated time periods, and the inhibitory rate was measured using an MTT assay. *n* = 3, mean ± SD. (**C**) Inhibitory effects of S5 on cell proliferation in human peripheral mononuclear cell. The cells were treated with 12.5, 25, 50, and 100 μM of S5 for the 24 h, and the inhibitory rate was measured using MTT assay. *n* = 3, mean ± SD.

To determine the cytotoxic effect, the viabilities of A375 cells treated with increasing concentrations and time of S5 were measured with an methylthiazolyldiphenyl-tetrazolium bromide (MTT) assay. It was found that S5 caused remarkable inhibition of A375 cell growth in a time- and dose-dependent manner. The IC_50_ value of A375 cells after treatment with S5 for 24 h was 36.88 μM (Figure 1B). However, the IC_50_ value of peripheral blood cells after treatment with S5 for 24 h was 82.99 μM (Figure 1C). The results suggest that S5 has significant anti-proliferation activity on human melanoma A375 cells, but has less toxicity to normal cells. The concentration of 40 μM was chosen for the subsequent experiments.

### 2.2. S5 Induces Cell Cycle G2/M Arrest in A375 Cells

**Figure 2:**
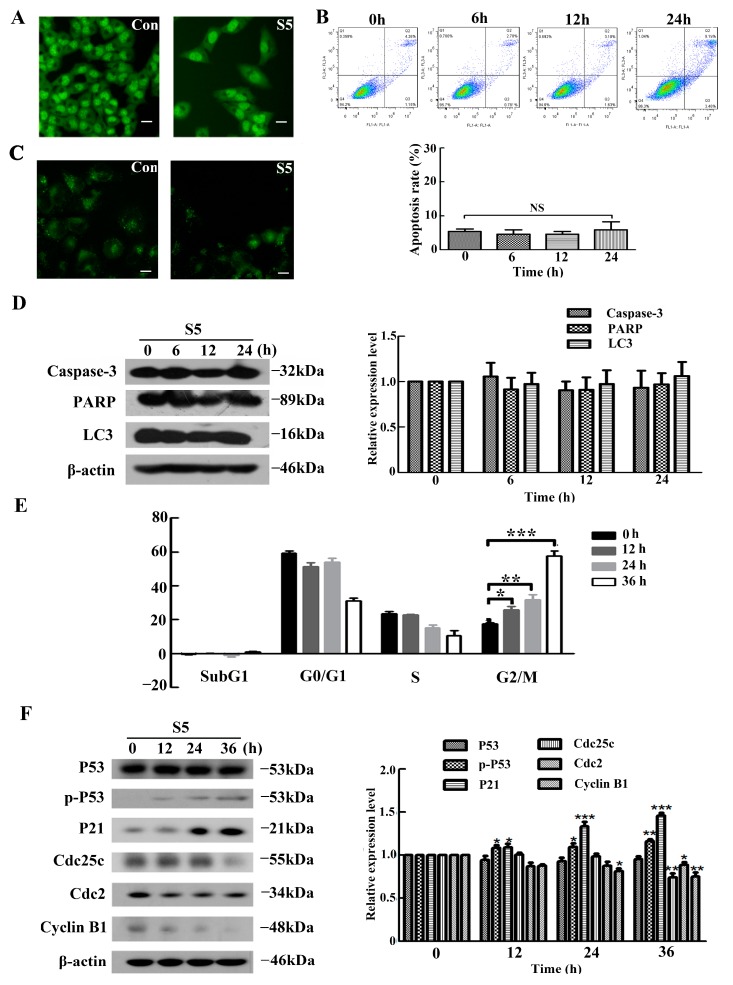
S5 induces G2/M arrest in A375 cells. (**A**) Nuclear morphology shown via AO staining of A375 cells treated with 40 μM of S5 for 24h. Bar represents 25 µm. (**B**) Induction of apoptosis was determined via staining with annexin V/propidium iodide (PI) and flow cytometric analysis. Values are expressed as mean ± SD. (**C**) The cellular morphologic changes were observed under fluorescence microscopy with MDC staining after being treated with 40 μM of S5 for 24 h. Bar represents 50 µm. (**D**) Western blot analysis of caspase3 LC3 and PARP expression levels in A375 cells after S5 treatments for 0, 6, 12, and 24 h. (**E**) The cells were cultured with 40 μM of S5 for 0, 12, 24, and 36h. The DNA content was analyzed using flow cytometry after PI staining. The percentage of cells in different phases of the cell cycle was represented using a bar diagram. Data from a representative experiment are shown. *n* = 3, mean ± SD. * *p* < 0.05, ** *p* < 0.01, *** *p* < 0.001. (**F**) S5 affected the expression of p-P53 and proteins involved in regulating the G2/M transition. The cells were lysed for protein extraction. Samples (25 μg) were subjected to 10% sodium dodecyl sulfate polyacrylamide gel electrophoresis (SDS-PAGE) and western blotting for the detection of specific proteins. All blots are representative of at least three repeats.

To further explore the mechanism of S5-induced cell death in A375 cells, we first investigated the effect of S5 on apoptosis. Acridine orange (AO) staining was used to estimate the changes in the cell nucleus (Figure 2A). The results of the fluorescence staining indicated that the number of living cells was reduced in A375 cells treated with 40 μM of S5 for 24 h. We also analyzed the apoptosis rate of A375 cells exposed to S5 using flow cytometry. As shown in Figure 2B, compared with the control group, S5 did not significantly increase the apoptosis ratio in A375 cells after treatment with 40 μM of S5 for 0, 6, 12, and 24 h. Furthermore, since autophagy also contributes to cell death, we investigated whether S5 induced autophagy in A375 cells. Autophagy is characterized by the increased acidic vesicular organelles, which are correlated with increased autophagosomes. We use monodansylcadaverine (MDC) label cellular acidic compartments, including lysosomes and autolysosomes. Upon exposure to 40 μM S5 for 24 h, the fluorescence intensity did not exhibit obvious increase in A375 cells (Figure 2C). Moreover, western blot was used to detect caspase3, PARP, and LC3. the maker proteins of apoptosis and autophagy. As shown in Figure 2D, the expression of caspase3, PARP, and LC3 did not change after treatment with S5 for 0, 6, 12, and 24 h. 

On the other hand, the analysis of the cell cycle phase distribution of A375 cells was assessed using flow cytometry. After treatment with S5 at 40 μM for 0, 12, 24, and 36 h, the rates of G2/M phase were 17.58%, 25.94%, 31.76%, and 57.50%, respectively (Figure 2E). Furthermore, cell cycle-regulating pathways were measured using western blotting. In Figure 2F, the expression of Cyclin B1, Cdc2, and Cdc25c, which play important roles in G2/M cell cycle progression [37], were markedly downregulated in cells after treatment with S5. Moreover, the expression of P21, one of CDK inhibitors [38], increased prominently in a time-dependent manner. Furthermore, the expression of phospho-P53 was also upregulated in a time-dependent manner by S5, while the expression of P53 remained unchanged. All these results demonstrated that S5 could not induce either apoptosis or autophagy in A375 cells, whereas it could induce cell cycle G2/M arrest in A375 cells.

### 2.3. ERK, P38, and EGFR Are Involved in the Anti-Proliferation Of S5 on A375 Cells

**Figure 3:**
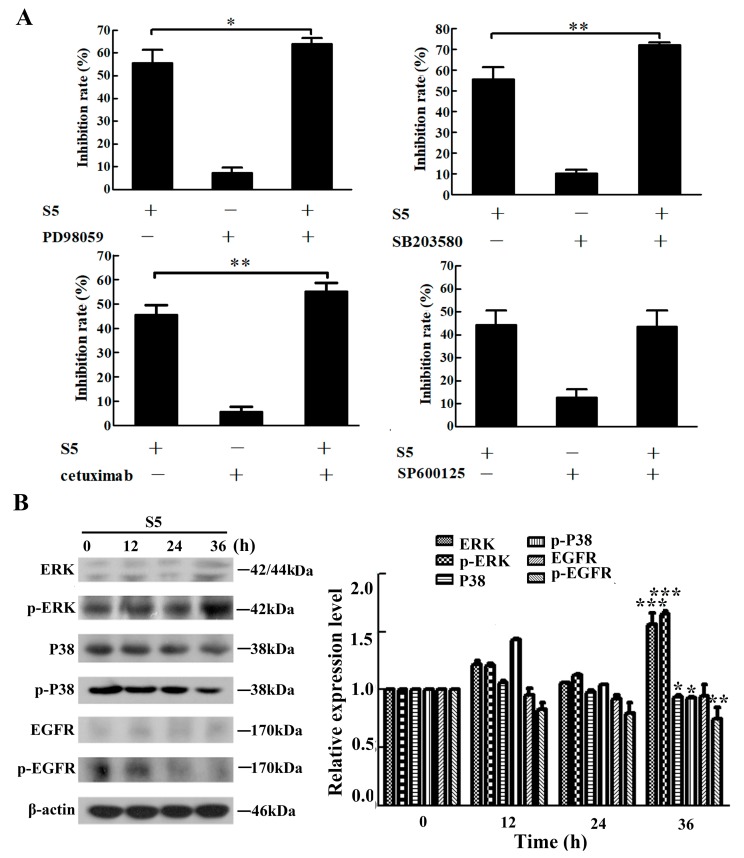
ERK, P38 and EGFR are involved in the anti-proliferation of S5 on A375 cells. (**A**) The cells were pretreated with 1.25 μM of SP600125, 5 μM of PD98059, and 5 μM of SB203580 or 40 μg/mL cetuximab for 1 h and then incubated with 40 μM of S5 for 24 h. The death rate of cells was measured using an MTT assay. (**B**) S5 affects the expression of MAPKs and EGFR proteins. The cells were lysed for protein extraction. Samples (25 μg) were subjected to 10% SDS-PAGE and western blotting for the detection of specific proteins. Results presented are the mean from three parallel experiments, *n* = 3, mean ± SD. * *p* < 0.05, ** *p* < 0.01, *** *p* < 0.001.

The mitogen-activated protein kinase (MAPK) pathway plays an important role in modulating cell growth and proliferation in melanoma [39]. In order to confirm whether S5 inhibits A375 cell proliferation via MAPK signaling pathway, we examined the effect of the JNK inhibitor SP600125, the ERK inhibitor PD98059, and the P38 inhibitor SB201580 on the S5-induced growth inhibition of A375 cells. As shown in Figure 3A, PD98059 and SB201580 both enhanced the inhibitory effect of S5 on A375 cells, whereas SP600125 did not affect S5-induced proliferation inhibition in A375 cells. 

Also, since the epidermal growth factor receptor (EGFR) is known to play a pivotal role in tumor growth [40], we investigated whether EGFR was associated with the anti-proliferative effect of S5 on A375 cells. The effect of an anti-EGFR monoclonal antibody cetuximab on the S5-induced growth inhibition of A375 cells was tested. The result (Figure 3A) showed that cetuximab could remarkably increase the inhibitory effect of S5 on A375 cells. 

Furthermore, the related proteins were measured using western blotting. As shown in Figure 3B, the expression of ERK and phospho-ERK were increased after treatment with S5 for 36 h, whereas P38 and phospho-P38 expression were decreased. The expression of EGFR did not change in S5-treated A375 cells, but the expression of phospho-EGFR was remarkably downregulated. Taken together, ERK, P38, and EGFR were involved in S5-induced cell growth inhibition on A375 cells.

### 2.4. Activation of EGFR/P38 Signal Transduction Is Associated with S5-Induced A375 Cell Cycle Arrest at G2/M 

**Figure 4:**
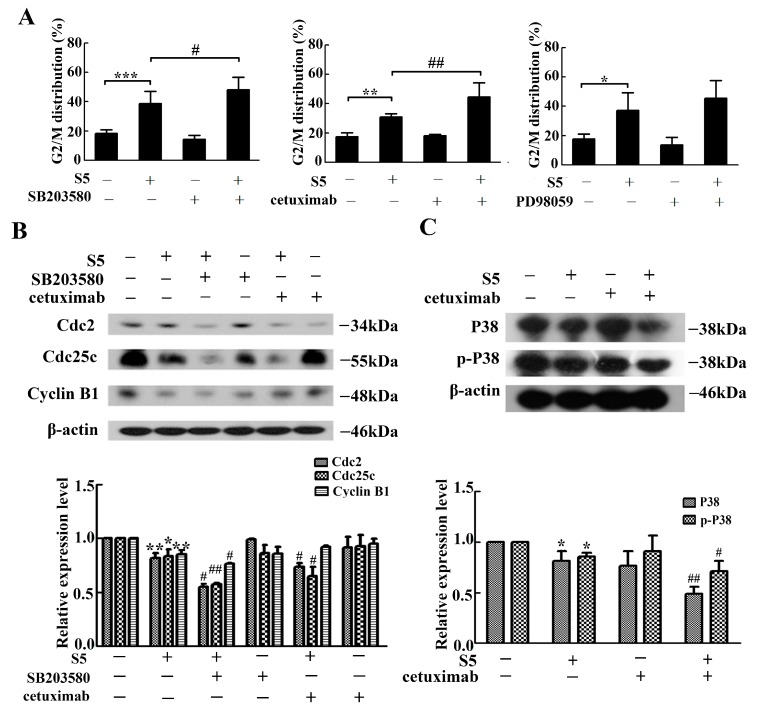
EGFR/P38 signaling pathway was involved in S5-induced cell G2/M arrest. The cells cultured with 40 μM of S5 for 36 h in the absence or presence of 5 μM of SB203580, 40 μg/mL of cetuximab or 5 μM of PD98059. (**A**) The percentage of cells in G2/M phase of the cell cycle was represented using a bar diagram. Data from a representative experiment are shown. *n* = 3, mean ± SD. * *p* < 0.05, ** *p* < 0.01, *** *p* < 0.001, # *p* < 0.05, ## *p* < 0.01. (**B**) The proteins expression of Cdc2, Cdc25c, and Cyclin B1 were tested using western blotting. * *p* < 0.05, ** *p* <0.01 vs. the control, # *p* < 0.05, ## *p* < 0.01 vs. the S5 treatment. (**C**) Cetuximab could further affect the expression of P38 and *p*-P38. The cells were lysed for protein extraction. Samples (25 μg) were subjected to 10% SDS-PAGE and western blotting for the detection of specific proteins. All blots are representative of at least three repeats. * *p* < 0.05 vs. the control, # *p* < 0.05, ## *p* < 0.01 vs. the S5 treatment.

We have already proven that S5 could induce cell cycle arrest at G2/M in A375 cells and also confirmed that S5 inhibits A375 cells proliferation via ERK, P38, and EGFR. To elucidate whether the ERK, P38, and EGFR contribute to the S5-induced cell cycle arrest at G2/M phase, the G2/M phase distribution and the expression levels of proteins that regulate the G2/M phase transition were measured after treatment with ERK and P38 kinase-specific inhibitors PD98059, SB203580, and anti-EGFR monoclonal antibody cetuximab. As shown in Figure 4A, S5-induced G2/M cell cycle arrest was markedly increased by SB203580 and cetuximab, while there were no significant changes when pre-treated with PD98059, suggesting that P38 and EGFR were involved in the cell cycle arrest at G2/M phase. To verify the above finding, we further tested the expression of several maker proteins of G2/M cell cycle arrest using western blotting (Figure 4B). It was found that the expression of Cdc2, Cdc25c, and Cyclin B1 were downregulated after treatment with S5. The P38 inhibitor SB201580 further intensified the downregulation of Cdc2, Cdc25c, and Cyclin B1, while the EGFR monoclonal antibody cetuximab could only further enhance the downregulation of Cdc2 and Cdc25c.

To confirm the relation between P38 and EGFR, cetuximab was used to detect the influence of EGFR on P38. As shown in Figure 4C, the expression of P38 and phospho-P38 were decreased after treatment with S5, and the pre-treatment with cetuximab further decreased their expression. These results suggested that S5 caused cell cycle arrest on G2/M through EGFR/ P38 signal transduction.

## 3. Discussion

A significant amount of literature has been reported stating that withanolides could exhibit significant anti-tumor effects on various tumors, including human head and neck squamous cell carcinomas [41], breast cancer [42], and liver cancer [43]. We previously also found that a variety of withanolides of *Physalis pubescens* L. have the effect of inhibiting renal carcinoma, prostate cancer, and melanoma cell proliferation [44]. S5 is one of active withanolide compounds derived from *Physalis pubescens* L. with antitumor effect [36]. Here, the aim of the present study was to assess the antitumor effects of S5 on human melanoma A375 cells. The results demonstrated that S5 has remarkable inhibitory activity on A375 cells, but it has lower toxicity on the peripheral blood cell. These properties are in agreement with second generation anticancer drugs, which exhibit selective cytotoxicity on tumor cells but relatively low-toxicity to normal cells [45]. Thus, S5 could be used as a potential agent in the treatment of melanoma. In particular, we found that S5 caused remarkable inhibition of A375 cell growth in a time- and dose-dependent manner, and the inhibition ratio of cells had a stationary phase after treatment with 36 μM to 54 μM of S5. The inhibition rate of A375 cell following treatment with S5 for 24 h in the stationary phase was about 50%, which indicated a good medicinal property of S5; thus, with appropriate adjustment, it can be used clinically. To explore the underlying mechanism of S5, its influence on apoptosis and autophagy in A375 cells was observed. Apoptosis and autophagy are the most common death modes caused by withanolides [42]. However, we found that there was neither apoptosis nor autophagy following treatment of A375 cells with S5, which was confirmed using both flow cytometry and western blotting. 

Ching-Yu Yen et al. have reported that 4β-Hydroxywithanolide E [46], as the withanolides, induced G2/M cell cycle arrest. In the current study, we also demonstrated that S5 can induce the G2/M cell cycle arrest in a time-dependent manner. Squatrito M et al. have pointed out that cells are engaged in complex events in response to DNA damage [47]. These events could result in the removal of the damage and also halt or slow down cell cycle progression until the damage has been repaired [48]. In this study, we also investigated the potential of S5 to reduce the expression of cyclin B1, Cdc2, and Cdc25c, but the action times were different, indicating that the mechanism of S5 in influencing the expression of cell cycle arrest proteins was more complex. Cuiting Lv et al. found that asperolide A leads to the inhibition of NCI-H460 lung carcinoma cell proliferation by G2/M arrest with the p53-dependent p21 pathway [49]. In addition, Yanlin Ming et al. also found that p-p53 and P21 expression level was upregulated while the Corilagin induced G2/M phase arrest on Hepatocellular carcinoma cells [50]. Similar to their results, we also found that S5 could increase the expression of P21 and phospho-P53. These results suggested that S5 further induced G2/M phase arrest through P53-P21 regulatory mechanism. The above results reflect that the antitumor potential of S5 could involve blocking the cell cycle progression between G2 and M phase, thus delaying the progression of the cells toward mitosis.

MAPK signaling pathway influenced the cell cycle progression and cell proliferation in melanoma [51,52]. In the current study, we demonstrated that the P38 inhibitor SB201580 markedly enhanced the cell inhibitory effect of S5 on A375 cells; meanwhile, S5 could decrease the expression of p-P38, suggesting that the P38 contributed to the anti-proliferative effect of S5 on A375 cells and has a pro-survival role. Additionally, the present results showed that inhibition of ERK could increase the cell inhibition rate of S5. However, when treated with S5 alone, the expression of ERK and p-ERK were raised. Furthermore, the results of our research demonstrated that ERK was not involved in S5-induced G2/M arrest of A375 cells. These results indicated that ERK had complicated functions in the proliferation inhibitory effect of S5 on A375 cells. The mechanism of how ERK influenced the cell inhibition needs to be further explored. The JNK inhibitor SP600125 did not have an obvious influence on A375 cells, indicating that JNK might be not associated with S5-induced A375 cell death.

Koprowski et al. described that EGFR is overexpressed in late melanoma [53]. A previous study showed that EGFR is involved in the anti-tumor effect of withanolide products [54]. In agreement with the report, we found that S5 can inhibit the phosphorylation of EGFR and the EGFR monoclonal antibody cetuximab could enhance the anti-proliferative effect of S5 on A375 cells markedly.

Some studies showed that ERK, P38, and EGFR all can take part in the G2/M arrest in cells [55,56,57]. In the present study, S5-induced cell cycle G2/M arrest was markedly increased by the P38 inhibitor SB203580 and the EGFR monoclonal antibody cetuximab, whereas there was no significant changes when pre-treated with the ERK inhibitor PD98059. Furthermore, the P38 inhibitor SB201580 and anti-EGFR monoclonal antibody cetuximab could further intensify the downregulation of Cdc2 and Cdc25c by S5. These results indicate that both P38 and EGFR were involved with in the G2/M arrest induced by S5 and played a reverse role. Interestingly, P38 inhibitor SB201580 could enhance S5-induced down-regulation of Cyclin B1, whereas cetuximab did not show a similar effect on the expression of Cyclin B1. Therefore, the p38 and EGFR signal pathways influenced the A375 cell cycle arrest differently and the phenomenon needs to be studied further.

MAPK activation is EGFR-dependent in most cells [58]. Qiang Dang et al. found that EGFR is upstream of P38 in the antiproliferative effects of kaempferol in renal cell carcinoma [59]. Other studies have also described that, to some extent, P38 is upstream of EGFR and it could modulate the expression of EGFR [60]. The relationship between EGFR and P38 is complicated and our results, in accordance with Qiang Dang et al., we demonstrated that the cetuximab could further decrease the expression of P38 and p-P38 protein, while the P38 inhibitor did not affect the expression of EGFR (data not shown), which suggested that EGFR might be one of the upstream of the P38 signal transduction pathway.

**Figure 5:**
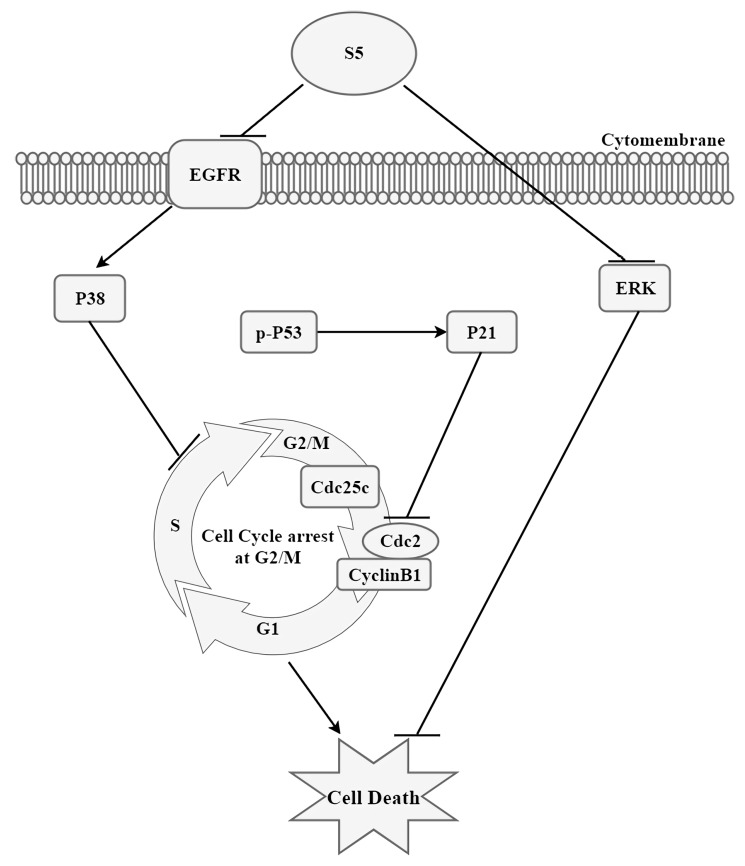
Schematic diagram of hypothesized mechanism of S5-induced death of human melanoma A375 cells.

In conclusion, our study, for the first time, identified the potential molecular mechanisms of the anti-tumor effect of S5 on melanoma cells. We found that S5 significantly induced G2/M cell cycle arrest via EGFR/P38 signaling pathway (Figure 5). This study showed that S5 is a novel anticancer drug candidate that has a great potential as a prospective agent in melanoma.

## 4. Materials and Methods 

### 4.1. Drug and Reagents

The purified (>98%) S5, an inseparable epimeric mixture, was identified as (20S,22R,24R,25S,26S)-15α-acetoxy-5,6β:22,26-diepoxy-4β, 24, 25, 26-tetrahydroxyergost-2-en-1-one and (20S,22R,24R,25S,26R)-15α-acetoxy-5,6β:22,26-diepoxy-4β, 24, 25, 26-tetrahydroxyergost-2-en-1-one, and it was generously provided by professor Feng Qiu (Tianjin, China) [36]. The ratio of two isomers of S5 in medium was R:S = 1:4. Stock solution of 100 mM was prepared in dimethylsulfoxide (DMSO) (Sigma, St. Louis, MO, USA) and stored at −20 °C. The high-glucose Dulbecco’s Modified Eagle’s Medium was obtained from Gibco/BRL (Gaithersburg, MD, USA). Fetal bovine serum (FBS) was obtained from Biological Industries (Bio-logical Industries, Kibbutz Beit-Haemek, Israel). The primary antibodies of p-P53, LC3, p-ERK, p-P38, and p-EGFR for western blotting were purchased from Santa Cruz Biotechnology (Santa Cruz, CA, USA). Also, rabbit polyclonal antibodies against PARP, P53, Cdc2, Cyclin B1, ERK, P38, and EGFR were purchased from Bioss Antibodies (Beijing, China). The antibodies against P21 and Cdc25c were purchased from ProteinTech Group (Rosemont, IL, USA). All horseradish peroxidase (HRP)-conjugated secondary antibodies (goat-anti-rabbit, goat-anti-mouse, and rabbit-anti-goat) were obtained from ZhongShanJinQiao company (Beijing, China). The ERK inhibitor PD98059, JNK inhibitor SP600125, p38 MAPK inhibitor SB203580, and other chemicals were purchased from Selleck (Houston, TX, USA). 

### 4.2. Cell Culture

The human melanoma A375 cells were obtained from the American Type Culture Collection (ATCC, Manassas, VA, USA). The cells were cultured in DMEM supplemented with 10% FBS, 10 µg/mL streptomycin, and 100 µg/mL penicillin, and maintained at 37 °C and 5% CO_2_ in a humidified incubator.

### 4.3. Cell Viability Assay

A375 cells were seeded onto a 96-well culture plates at a density of 6 × 10^3^ cells/well for 24 h and then treated with various concentration of S5 (16, 24, 36, 54, and 81 μM). After 6, 12, 24, 36, and 48 h, the cells were washed using PBS, then MTT was added to a final concentration of 0.5 µg/mL, and the cells were further incubated for 2.5 h. The resulting crystals were dissolved in DMSO. The optical density (OD) was measured at 490 nm using a microplate reader (FlexStation 3, Molecular Devices, CA, USA). The percentage of viable cells was calculated according to previous report [61]. 

The human peripheral blood cells were seeded onto 96-well culture plates at a density of 15 × 10^5^ cells/well for 3 h and then treated with various concentration of S5 (12.5, 25, 50, and 100 μM). After 24 h, the percentage of cell toxicity was measured using an MTT assay.

The A375 cells were seeded onto 96-well culture plates at a density of 6 × 10^3^ cells/well for 24 h and pretreated with or without 1.25 μM of SP600125, 5 μM of PD98059, and 5 μM of SB203580 or 40 μg/mL cetuximab for 1 h, and then incubated with 40 μM of S5 for 24 h. Viability of A375 cells was assessed using an MTT assay.

### 4.4. Fluorescence Microscopy Examination

The apoptotic and autophagy morphology were assessed by staining the cells with the fluorescent DNA-binding dye acridine orange (AO) and monodansylcadaverine (MDC) separately. After treatment with S5 for 24 h, A375 cells were stained with 20 µg/mL AO or MDC for 15 min, and finally the nuclear morphology was observed under a fluorescence microscope (Olympus, Tokyo, Japan). 

### 4.5. Flow Cytometric Analysis of poptosis and Cell Cycle Distribution

Cell apoptosis was analyzed by a combined staining of annexin V and propidium iodide (PI) using the FITC Annexin V Apoptosis Detection Kit (BD Biosciences, San Diego, CA, USA). After treatment with 40 μM of S5 for 0, 6, 12, and 24 h, A375 cells were washed with binding buffer and centrifuged. The cell pellets were resuspended in binding buffer and 5 µL Annexin-V and 5 µL PI were added, mixed, and the preparations incubated for 15 min in the dark at room temperature. The apoptotic cells were measured using a BD FaCSCalibur flow cytometer (Becton-Dickinson, Franklin Lakes, NJ, USA). 

To evaluate the cell cycle distribution, A375 cells were seeded in six-well plates with a density of 7.5 × 10^4^ cells/mL and then treated with S5 at 40 μM concentrations for 0, 12, 24, and 36 h or treated with or without PD98059, SP600125, or SB203580 at the given concentrations for 1 h and subsequently treated with S5 for 36 h. After S5 treatment, the cells were harvested, washed with phosphate-buffered saline (PBS), and added the 10 mL cold ethyl alcohol dropwise, maintained at −20 °C overnight. Then, the cell pellets were stained by 1 mg/mL PI, which contains 50 μL RNase on ice in the dark for 30 min. The cell cycle distribution was measured using a BD FaCSCalibur flow cytometer (Becton-Dickinson, Franklin Lakes, NJ, USA). All data were recorded and analyzed using the FlowJo software version 7.6 (Tree Star, Inc., Ashland, OR, USA). 

### 4.6. Western Blot Analysis

Cells were seeded in six-well plates at a density of 1.5 × 10^5^ cells/mL. After 24 h of incubation, they were treated with or without PD98059, SP600125, or SB203580 at the given concentrations for 1 h and subsequently treated with 40 μM of S5 for different time periods. Then both adherent and floating cells were collected, and then lysed in ice-cold RIPA for 30 min on ice. Cell lysates were centrifuged at 12,000 r/min for 15 min at 4 °C and the supernatants were collected. Protein concentrations were quantified using the BSA Protein Assay (Solarbio Life Sciences, Beijing, China) according to the manufacturer’s instruction. Equal amounts (30 μg) of total protein were separated using SDS-PAGE (10–12%) at 70 V for 1.5 h and transferred to a PVDF membrane at 100 V for 2.5 h. The membranes were blocked with 5% non-fatty milk in TBS/0.5% Tween 20, and then indicated with primary antibodies at 4 °C for overnight. After three washes with TBS/0.5% Tween 20, anti-mouse, anti-goat, or anti-rabbit IgG was incubated with the membranes for 2 h at room temperature. Signals were developed using an ECL western blotting detection kit (Sigma Chemical, USA) and exposed to X-ray film. 

### 4.7. Data Analysis

All data represent at least three independent experiments and are expressed as the mean ± SD. These data were analyzed using IBM SPSS statistics 17.0 (SPSS, Chicago, IL, USA) software. One-way ANOVA was employed to calculate the significance between sets of data. For all analysis, *p* < 0.05 was considered to indicate a statistically significant result.
